# MR spectroscopy in HIV associated neurocognitive disorder in the era of cART: a review

**DOI:** 10.1186/s12981-021-00388-2

**Published:** 2021-10-09

**Authors:** Joga Chaganti, Bruce James Brew

**Affiliations:** 1 St Vincent’s Hospital, Sydney, Australia; 2 Department of Medicine (Neurology), University of New South Wales, Sydney, Australia; 3 Department of Medicine, Head Neurosciences Program and Peter Duncan Neurosciences Unit, St Vincent’s Centre for Applied Medical Research, President of the International Society for Neurovirology 2019, University of Notre Dame Sydney, Sydney, Australia

**Keywords:** HIV, HAND, Neurocognitive impairment (NCI), Proton MR spectroscopy (MRS)

## Abstract

Neuroimaging has been a critical tool for understanding the neuropathological underpinnings observed in HIV. The pathophysiology of HAND is chiefly driven by neuroinflammation. Despite adhering to cART, low levels of viraemia probably persist in the brain in some patients leading to chronic immune activation with resultant neuroinflammation and consequent neuronal injury. MR spectroscopy has been widely used as a biomarker for the presence and severity of HAND in several studies. By studying the MRS signatures, it is possible to characterise the presence of neuroinflammation and neural injury. Furthermore, metabolite concentrations measured by MRS could be used as a quantitative indicator of HIV cerebral involvement, thereby affording the opportunity to assess the efficacy of cART in HAND. However, currently there are three significant limitations in the MRS HIV research literature: the relative paucity of prospective studies, the small number of regions of interrogation due to current methodology (single voxel MRS), and the evolving understanding of the impact of co-morbidities (e.g. ageing, mood disorders, alcoholism etc.) on MRS measurements. This review critically addresses the current literature of MRS studies in people living with HIV (PWH) with HAND to determine its value, especially in the context of the current cART era. In addition, we discuss technical considerations related to the disease and the future direction in HAND using MRS.

## Key points

1. MRS is a reproducible surrogate marker to measure the brain injury in HIV.

2. In the cART era, the neurocognitive impairment is characterised by increased Ch and Mi in frontal white matter and basal ganglia early on and in the later stages there is compromise in the neuronal integrity signalled by reduced NAA.

3. Creatine concentrations change with severity of dementia and therefore metabolite concentrations should be expressed either as absolute values or relative to internal water.

4. MRS changes can take as long as 6–12 months to correlate with the neuropsychological improvement (although paradoxical, this is a significant observation in two respects: that the cellular level changes take longer time to both clinically manifest as well as takes longer time to revert back in spite of apparent clinical improvement).

5. Glx compounds appear to be more sensitive to identify the early neurocognitive impairment and early indications are that the excitotoxic pathway can help to identify the treatment response as well.

6. MRS complements the other advanced imaging measures in the assessment of the influence of the co-morbidities on cognition.

7. With the emergence of the newer evidence in the development of HAND, the metabolites Glx and GABA are likely to play a key role in the early diagnosis as well as treatment response in patients with HIV associated neurocognitive disorders.

## Introduction

The diagnosis of HAND in the era of cART can be challenging. Currently HAND is best diagnosed by NC testing which requires trained staff. However, quantitative imaging techniques are emerging as a potential alternative. MRS is a robust technique which measures the chemical environment in the brain and offers insight into the neuronal integrity, cell membrane synthesis and turnover, inflammation status, and levels of microglial activation and astrogliosis within the sampled CNS tissue. The concentration of these neurochemicals can then be measured from the area under the curve of that particular spectral peak.

Given the very significant effect of cART on HIV disease, the literature will be reviewed in two sections: pre-cART period (prior to 1997) and the era of cART (after 1997), after a brief synopsis of the technical aspects of MRS.

### Technical considerations

The principle behind the MRS technique is suppression of the water signal and extraction of metabolites which have differing resonance frequencies; these can be separated from each other using Fourier transformation into different spectral peaks (1–5 ppm range). The concentration of these neurochemicals can then be measured.

The measured metabolites include NAA, Cho, Cr, Mi, Glx and GABA. The resonance of the metabolites is not uniformly seen on all MR acquisition sequences and is dependent upon the TR and TE. The short TE sequences capture the resonance of the metabolites with short relaxation times (eg. Mi and Glx, GABA as well as free lipids). The NAA, Cr and Cho resonance can be captured on both long and short TE sequences.

NAA is found predominantly in neurons and is known to be present at concentrations of 9–12 mmol in neurons, and as such is regarded as a marker of neuronal density. In addition, NAA is believed to play several roles: as a reserve for energy metabolism, a supply of acetyl CoA, a source of glutamate and an important role in osmoregulation. NAA is synthesized in the mitochondria of neurons and therefore mitochondrial dysfunction is known to cause reduced NAA. Neuronal cell death leads to irreversible loss of [1] NAA while mitochondrial dysfunction in neurons leads to reversible loss of NAA [1].

Cho is a membrane marker whose elevation reflects membrane inflammation and myelin breakdown. Cho mainly consists of glycerophosphocholine and phosphocholine, compounds involved in phospholipid metabolism in brain tissue. Cho is present in the cell membrane of all cells; however, it is more abundant in glial cells. The resonance is attributed to trimethyl ammonium residues of free choline, phosphorylcholine, glycerophosphorylcholine and other metabolites such as carnitine. Cho is typically higher in the white matter than in gray matter [2].

Mi is present almost exclusively in glial cells and as such is a putative marker for glial cells [3]. It acts as an osmoregulator and is increased in any process that causes glial activation. The brain and cerebrospinal fluid (CSF) are relatively enriched in Mi compared to plasma, with estimated typical concentrations of 6 mM in brain, 0.2 mM in CSF, and 0.03 mM in plasma [4]. Within the brain Mi is an intracellular molecule. It is taken up into cells via two sodium-Mi cotransporters, SMIT1 and SMIT2, and a hydrogen-myo-inositol symporter, HMIT. SMIT1 and SMIT2 are expressed by both neurons and glia, although SMIT1 is predominantly astrocytic and SMIT2 predominantly neuronal [5]. Mi concentrations appear generally to be higher in glia than neurons, and glial uptake of Mi exceeds that of neurons, possibly due to expression of the HMIT transporter.

Cr is a complex of amino acids and is present in most of the cell types in the brain. It is usually regarded as relatively constant in the brain parenchyma though there are some disease states where it may be reduced [6, 7]. CrT (total creatine) is the concentration of Cr and PCr (phosphocreatine) in the brain tissue. It is utilised as an energy reservoir in cells with high energy demands as it is a part of creatine kinase energy metabolism buffer system used to maintain ATP levels. Glial cells have a four times greater concentration compared to neuronal cells [1].

**Fig. 1:**
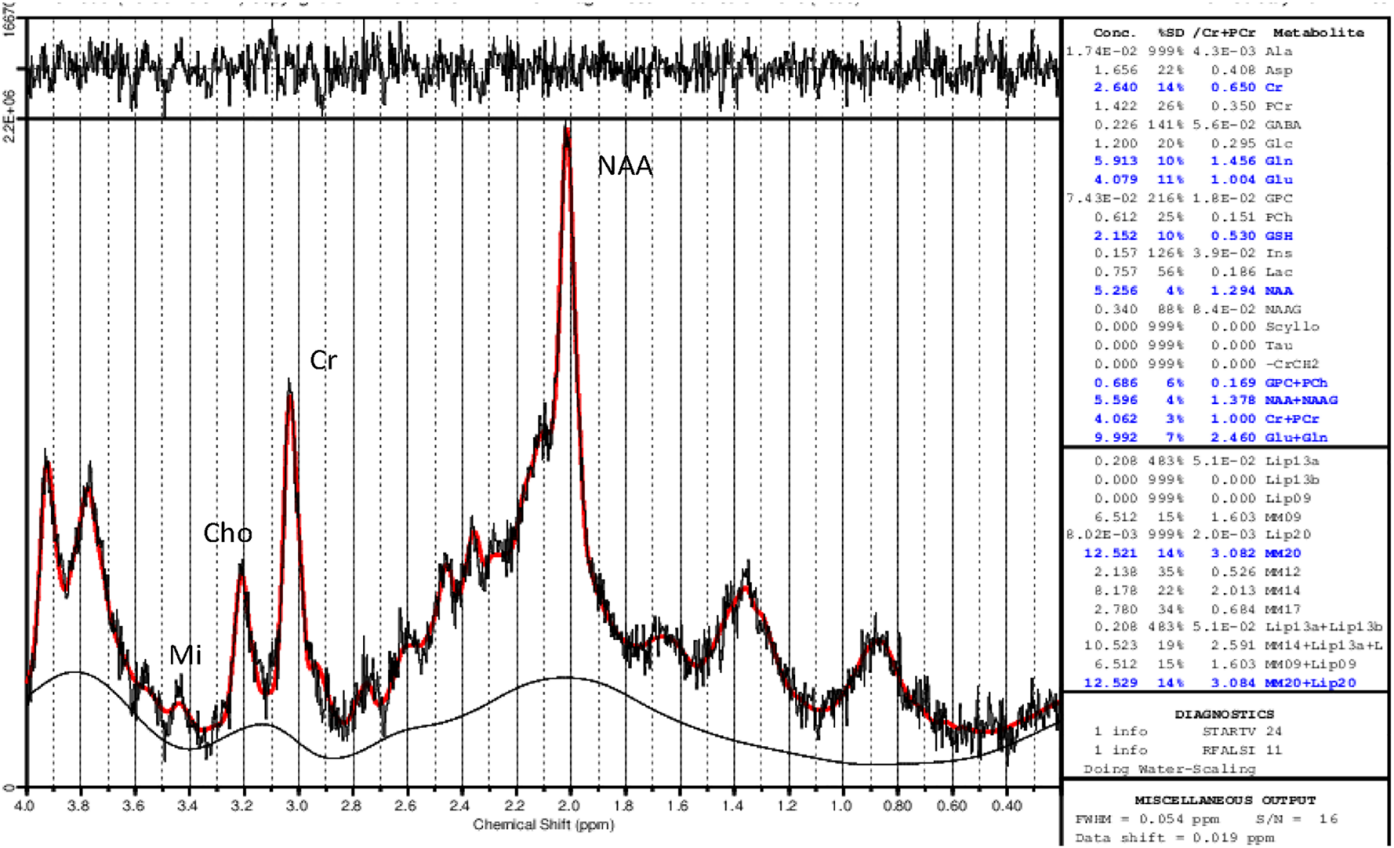
Normal MR spectrum from FWM demonstrating normal metabolite concentrations (internal water as standard)

Glx is a unitary term for two excitatory amino acids (glutamate and glutamine) involved in normal neuronal communication; raised levels are associated with excitotoxicity [8]. GABA is a neuroinhibitory amino acid potentially involved in the disrupted default neuronal network associated with impaired memory as a compensatory response to excitotoxicity [8] (Fig. 1).

Metabolites’ measurements are not only dependent on the acquisition methods but also on editing techniques. Newer sequences such as 2D L-COSY (two dimensional (2D) localised chemical shift correlated spectroscopy) is an acquisition technique that uses several additional frequency applications to detect the molecular resonance that otherwise is undetectable in 1D-1H MRS. MEGA-PRESS uses a post processing editing technique to unmask the spectra which otherwise cannot be detected due to a stronger resonating molecule at similar resonating frequency (eg. GABA and total creatine) utilising their unique spectral characteristic of J-coupling (GABA) [9].

**Fig. 2:**
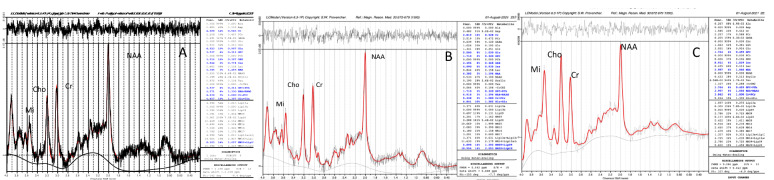
Representative MR Spectra in the FWM in showing progressive increase in Mi, Cho and reduction in NAA [from ANI(A)-MND (B) and HAD(C)] measured as internal water as standard

The metabolite values derived from MRS are often expressed as a ratio with reference to creatine as an internal standard. However, Cr has been shown to vary with age [6, 7] as well as the severity of HAND [10], potentially leading to inaccurate results. Therefore, internal water has been used as a standard given that it is more robust and reproducible (Fig. 2).

Initial MRS reports were either long TE single voxel PRESS studies [10–12] or long TE Multivoxel techniques in HIV Dementia patients [13–15]. The long TE techniques attenuate the signal from many metabolites considered important in the measurement of HIV associated brain injury (the compounds such as Mi, Glx and GABA cannot be measured on long TE sequences). As a result, long TE PRESS (point RESolved spectroscopy) studies were replaced by short TE PRESS methods. While short TE PRESS can show metabolites that reflect brain injury associated with cognitive impairment, this technique also has limitations, chiefly the small area of interrogation. Furthermore, the metabolites have overlapping spectra and require specialised post processing techniques for separation such as GABA and creatine and Glt and Gln.

### Pre-cART era

Whilst the pre-cART era is a matter for history for most populations its characteristics are still relevant and instructive. Pre-cART findings in MRS can provide a backdrop for the efficacy of cART and can serve as a guide to the MRS findings in cART naïve patients as well as those who have multiresistant HIV.

However, studies in this period were often limited by the data acquisition and analysis methods. Long TE sequences were dominantly used hindering identification of Mi and did not specifically target areas known to be vulnerable in HAD [13, 15, 16]. Jarvik et al. moved the field forward by using steam sequence (STimulated Echo Acquisition Mode-STEAM) (Short TE) to study the proton MRS (Hydrogen proton MR spectroscopy) role in HIV induced neural injury in the areas of abnormal appearing white matter and compared these to normal areas/centrum semiovale [17]. They showed that there are significant differences: reduced NAA/Cr, increased Cho/Cr, increased Mi/Cr ratios in HAD patients and an increase in a marker peak/Cr (this peak identified between 2.2 and 2.4 and likely Glx). Initial studies in the pre-cART period showed the Cho/Cr ratios were abnormal in HIV infected patients regardless of neurocognitive impairment and were seen in most parts of the brain (FWM, BG, Centrum Semiovale and FC [10, 11, 17, 18]. The Cho elevation was postulated to be due to either increased cellularity (increased macrophages and microglia) or increased cell membrane break down or both. The putative glial marker Mi was abnormal as a ratio to Cr in various stages of HAND [19, 20] and shown to be proportional to the dementia severity in the FWM [19]. The NAA/Cr levels were reduced in cognitively impaired while asymptomatic seropositive patients were shown to have normal NAA/Cr ratios [11, 17, 19, 20] [Jarvik et al.: Abnormal white matter, Laudenberger et al.: abnormal white matter and bilateral occipital cortex, Tarcey et al.: parietal midline white matter and Chang FWM, FC and BG). Advancing dementia was correlated with reducing NAA/Cr [12] (Mayeroff et al. 1993-Multivoxel short TE above the corpus callosum].

However, later studies were not uniform in this observation of normal NAA/Cr in cognitively unimpaired subjects as compared to the earlier studies [10, 21, 22]. These differing observations are most likely due to the use of Cr as internal standard. Cr is known to be in higher concentrations in astrocytes than in neurons. Astrogliosis, a known histopathological sequel in chronic HIV patients would lead to increased Cr on MRS. (Cr was shown to increase in the frontal white matter while reduced in the basal ganglia in HAD group). Studies in animal models have shown that the levels of Cr do not remain stable in HIV associated brain injury [8, 23]. As Cr concentrations could change with dementia severity, metabolite concentrations rather than metabolite ratios should be measured [23].

In summary in the pre-cART era the characteristic findings on MRS are reduced NAA/Cr and NAA/Ch and elevation in Choline and Myoinositol in cognitively impaired patients while those who were asymptomatic showed proportional elevation in Choline and Myoinositol only.

### Global changes in the pre cART era

**Table:** 

Dementia (CN/FWM)	↓ NAA	↑ Mi	↑ Cho
MCMD (FWM)	± ↓ NAA	↑ Mi	↑ Cho

### MRS in cART era

“*The overarching findings in the era of cART in PWH, are reduced neuroinflammation and reduced neuronal loss, which are reflected in normalised or reduced elevation of Cho and Mi and normalised or low normal NAA when compared to the normal controls *[22]”. These findings also correlated well with improvement in CD-4 counts and neuropsychology performance [18, 24]. However, these observations were not uniform with several later studies showing improvement but not necessarily normalisation with cART [21, 22, 25]. These inconsistencies have subsequently been addressed by using longitudinal studies.

### Longitudinal studies

“*Several of the longitudinal studies have shown that there is spectroscopic improvement with institution of cART*” [18, 24, 26, 27]. Some of subsequent studies have shown that these changes persist in chronically infected patients despite restoration of immunological status and effective viral suppression in response to antiretroviral therapies [28–30].

These varied results in metabolite ratios in multiple studies are likely multifactorial and driven at least partially by expression of the metabolite concentrations as ratios relative to Cr [23]. In addition, there are likely several pathological processes driving neural injury as patients are living longer and developing various comorbidities as well as differing degrees of response to cART [31].

Some longitudinal studies have revealed improvement in the metabolite ratio of NAA/NAA + Cr + Cho compared to the baseline in the first few months of treatment (↑ NAA) [26, 27]. Chang et al. demonstrated significant improvement in the metabolites (Choline compounds elevated at 3 months in FWM, FGM, BG and continued to be elevated even at 6 months but showed reversal after 9 months) of the HIV + cohort after 6–9 months, while improvements in CD4 cell counts and viral loads (measured in the plasma and CSF) occurred after 3 months of treatment [18, 23]. Tarasow et al., also demonstrated similar improvements in neuronal integrity (increased NAA/Cho ratios) approximately after 6 months in FWM and BG [25]. Both these studies also demonstrated that the spectral improvement correlated with clinical improvement in multiple domains (Improvement in neurocognition: Chang et al., CD-4 counts: Wilkinson et al., HIV encephalopathy) [19, 27]. The early reversal of metabolites in some of the initial studies [24, 26] was likely to be due to the lower creatine ratios at the base line due to treatment effect at the base line cohort [21].

However, the improved metabolites concentrations were later found to be inconsistent in some of subsequent studies. Salvan et al. [10] have shown that the improvement was only observed in those patients who had decreased NAA/CR at baseline while those who had elevation in the Cho/Cr did not improve much [10, 13]. Some studies, also showed persistent elevation of Mi, but not Cho, in the FWM, suggesting ongoing neuroinflammation with glial activation despite viral suppression. Nevertheless, the degree of elevation was less than that observed in cART-naïve HIV patients [23, 32].

More recently, two studies have explored the longitudinal effects of patients with HIV infection and response to cART. Young et al. [33] recruited fifty-three participants at a median of 3.7 months post HIV transmission and followed for a median of 6.0 months. They observed increases of Cho/Cr and MI/Cr in the frontal white matter and parietal gray matter as well as Glu a marker of excitotoxicity, in the basal ganglia in treatment-naive patients which reduced after initiation of cART [33]. Sailasuta et al. have used single voxel MRS in four brain regions (FWM, FGM, BG, PC) in cART-naïve subjects before (n = 59, 27 with HAND) and after 12 months of cART. The salient observation was Persistent elevation of Cho was noted in individuals who remain impaired after 12 months of cART in the posterior cingulate (PC) [34]. This cohort of cognitively impaired group also exhibited decreased glutamate in both FWM (P = 0.027) and BG (P = 0.013) as compared with those without HAND. This study has raised an interesting association of Choline but not myoinositol in patients who were on stable therapy with NCI similar to earlier study by Chaganti et al. [35].

Three recent studies have explored the interactions of cART and neurometabolites in virally suppressed people living with HIV in a longitudinal design. Cysique et al.; in their longitudinal design (23 month ± 5) (areas of interrogation: FWM, BG, PCC using water as an internal standard) in an aviremic cohort have observed progressive reduction in creatine in the FWM and PCC in stable HAND and a sharper decline in progressive HAND [36]. Other metabolites (however, this study showed reduced baseline NAA in CA and PC and increased Mi in PCC) did not show any significant change over time in this cohort with stable neurocognition [33]. Boban et al. used a longitudinal design to study an aviremic cohort using 2D CSI with short TE in a 5 year follow up and interrogated prefrontal cortices, anterior and PCC, intraparietal sulci, and frontal centrum semiovale white matter; the ratios of creatine to NAA, Cho and Mi were assessed. “*Although this study demonstrated significant increase in the NAA/Cr ratios in those who showed NC improvement, it has used creatine as the internal standard which may confound the outcomes*” [37]. In this study, none of the other metabolites revealed any consistent changes across the brain regions interrogated.

The improvement of NAA metabolite concentrations is interesting and is most likely due to one of the mechanisms detailed below. The role of NAA in myelin lipid synthesis, particularly in early development, is well established. The acetic acid from NAA becomes incorporated into CNS myelin [38]. Under metabolic stress, a shortage of acetyl-CoA could result in reduced NAA synthesis and increased hydrolysis of NAA to provide acetate for myelin repair [38] It is therefore likely that there are several mechanisms in play in the increase in NAA after treatment. 1: The creatine reduction in patients on cART when measured as a ratio to creatine. 2: Correction of transient depression of synthesis due to reduced availability of acetyl-CoA and 3: Reduced NAA hydrolysis and neuroplasticity could play a significant role in improvement of NAA.

Another longitudinal study of interest is from Gongvatana et al. They explored two groups; HIV on stable cART and NAS and followed them over two years. This study explored the midfrontal cortex (MFC), FWM, and BG and found that the HIV-infected subjects showed significant annual decreases in brain metabolite levels in all regions examined, including NAA (2.95%) and Cho (2.61%) in the FWM; NAA (1.89%), Cr (1.84%), Cho (2.19%), and Glx (6.05%) in the MFC; and Glx (2.80%) in the BG. They further identified that the neurocognitive decline was associated with longitudinal decreases in Glx in the FWM and the BG, and in NAA in the BG. “*This study concluded that there are widespread progressive changes in the brain in chronically HIV-infected persons despite stable antiretroviral treatment and virologic suppression and can lead to neurocognitive declines*” [39].

“*Putting these studies together, the outcome measures based on metabolite improvement could take longer and may not entirely dependent upon the antiretroviral therapy alone. Other factors such as comorbidities and the nadir CD4 counts, presence of NCI, and limited efficacy of cART or potential cART neurotoxicity *[40] *may influence the final outcomes. In patients who are aviremic, although there are no conclusive observations, there are early indications to suggest that there is a trajectory of metabolites with normal NAA and persistently increased choline and variable myoinositol*”.

### Regional specificity of metabolites and relationship to the neurocognitive function in PWH

Several studies in the cART era in PWH have shown that there is persistence of metabolite abnormalities despite restoration of immunological status and effective viral suppression in response to antiretroviral therapies [28–30].Common areas of interrogation are include FWM, FGM, BG and in some instances the parietal white matter, parietal gray matter and cingulate.

The most common spectral abnormalities in MND are those of marginally elevated Ch/Cr, MI/Cr in the frontal white matter and frontal cortex [39] whereas in those with dementia (HAD), these metabolite ratios were elevated in both in the basal ganglia and frontal white matter with reduced NAA [19, 40]. However, single voxel MRS studies suffer from the limited areas of interrogation and a global understanding of the extent of this injury is therefore limited*.* To overcome this limitation multivoxel MRSI was employed [22, 27, 41]. In their study with short TE, Lopez-Villages observed elevated MI/CR in the frontal white matter in NAS and only decreased NAA/CR and normal MI/CR in gray matter of patients with HAND [41].

A recent study by Boban et al. using long TE MRSI demonstrated that the impact of the HAND is more widespread low NAA/Cr ratios in the cingulate gyrus and subcortical frontal and parietal and deep frontal white matter in the chronic aviremic group. In the other two groups chronic HIV untreated and normal controls, Mi increased in the FWM as well as AC in the untreated group. However, it’s important to note that this study only evaluated the blood but not CSF to characterise the aviremia and used the creatine as reference standard to measure the metabolites [37].

**Table:** 

PWH cf to Normal (Global Metabolites)	↓ NAA	± ↑ Mi	↑ Cho

### MRS and neuropsychology measures

#### MRS showed a positive correlation with neurocognitive tests and hence can provide a positive complimentary information

The International HIV Dementia Scale (IHDS) was developed to screen for HIV-associated dementia (HAD), but it has been used more generally for HIV-associated neurocognitive disorder (HAND). These batteries of tests comprise of Trail Making Test A, WAIS-III Digit Symbol (DS) and HVLT-R Total Recall and DS, BVMT-R Total Recall and Grooved Pegboard Test-Dominant Hand. The objective is to study the functional domains that are most likely to get impacted in HIV [42].

HIV patients may show significant neurocognitive improvements in psychomotor speed as early as 6 months after cART [43]. However, the limited by availability of trained staff as well as practice effects makes the routine use of neuropsychology tests difficult to implement. Neuropsychological improvement has been shown to correlate with MRS changes thereby overcoming the latter mentioned difficulties with neuropsychological evaluation.

There have been several studies that have described the metabolic substrates underlying more detailed neuropsychological performance and these studies broadly indicate that the neuropsychological impairment is associated with markers of neuronal damage and increased markers of gliosis in the basal ganglia and frontal white matter.

Studies revealed a strong positive correlation between measures of gross and fine motor function and NAA/Cr ratios in the FWM (frontal white matter) and negatively with MI/Cr in the basal ganglia [41, 44]. Similarly, cognitive processing speed was negatively correlated with MI/Cr in the basal ganglia [23, 45, 46] and frontal lobe dysfunction (as defined by neuropsychology) with Mi [23, 41]. The ACTG 301/700 trial assessed changes in the NPZ-8 score (a summary score of a brief battery of neuropsychological tests) and MRS markers in the context of a trial of the efficacy of memantine in HAND. Improvement in NAA/Cr but not NPZ-8 scores was observed at 16 weeks of therapy [47].

*“It’s likely that MRS can play a role in the early detection of HAND, in determining its progression, and in assessing response to therapeutic interventions”. *[31].

### Early HIV infection and MRS

Interesting research in early HIV infection by Lentz et al. (early was defined as HIV seroconversion and imaged within 60 days of an evolving Western blot, while still having detectable plasma virus) demonstrated neuronal dysfunction soon after infection: “*reduced NAA and Glx in the cortical grey matter*”*.* They correlated the MRS markers with T-Cell phenotypes and found reduction in NAA and Glutamates in the frontal cortex [48]. These findings correlate with another similar study using volumetric and DTI analysis in the first 100 days of HIV infection wherein they demonstrated that there are volumetric (loss of parenchymal volume) and diffusion changes. This study contributes to the postulation that the initial early inflammation secondary to unchecked viremia may lead to significant neural injury. Furthermore, the focality of these findings suggests intrinsic brain inflammation but the effect of systemic inflammation is still possible.

### MRS and other imaging correlates

Volumetric imaging, DWI techniques and Single Photon Emission Computerized Tomography (SPECT) studies have been found to correlate with MRS derived metabolites and neurocognitive scores. However, volumetric measures and SPECT perfusion techniques appear to be less sensitive than MRS, while DWI and fMRI appears to show equal sensitivity overall.

### Volumetry and MRS

For volume loss to occur, there should be significant neuronal and axonal loss. It is therefore not surprising that MRS is more sensitive than volumetry to identify cognitive dysfunction (Paul et al. 2008: Caudate volumes and cognitive function) [49]. Further analyses have shown that NAA reduction correlates with total cortical volume loss while increased choline correlated with increased brain volume (possibly related to inflammation and oedema). Glx, osmoregulator and excitotoxic ion, is notable for its close relation to subcortical structural volumes [29].

### SPECT and MRS

Ernst et al. studied the comparative sensitivity of the SPECT derived blood flow and the metabolite concentrations in patients with HAND. While the SPECT studies showed some trend in decrease in the rCBF in the temporoparietal white matter, MRS was abnormal in multiple areas (reduced Cr concentration in the BG and increased Mi concentrations in the BG and the temporoparietal white matter) pointing to the superiority of MRS [50].

### DWI and MRS

Diffusion imaging explores the function of Brownian motion of the hydrogen proton in the CNS environment which allows assessment of the micro-architectural detail of white matter tracts and its integrity. DWI MR metrics showed strong positive correlations with glial metabolites. The increased diffusion (mean diffusivity) is thought to be associated with increased glial activation and inflammation [51]. The relationship of the metabolites and the treatment responses were also correlated with DTI (Diffusion Tensor Imaging) metrics FA (Fractional Anisotropy) and MD (Mean Diffusivity). Further, one study of Lithium as a treatment showed improving neuropsychology scores corresponded with an increase in the FA and reduction in MD along with reducing levels of the neuronal metabolite complex Glx [52].

### Functional MRI (fMRI) and MRS

*Gliosis associated with neuroinflammation plays a crucial role in influencing the patterns of fMRI. The striato-frontal cortical and subcortical involvement in HIV influences working memory impairment and is reflected in fMRI.*

The Blood oxygen dependent neurovascular coupling response that is associated with any motor task leads to local tissue level perfusion changes which is exploited in fMRI. fMRI has been extensively investigated in HAND to assess different neurocognitive domains with specific tasks. The working memory network which comprises the posterior parietal cortex and lateral prefrontal cortex were correlated with neurometabolites. The glial metabolites Mi, Ch and Cr-T in the basal ganglia and frontal white matter were positively correlated with increasing loads on the working memory derived signal strength [53]. This study also observed that the working memory network was not correlated with the concentration of NAA, which are markers of neuronal viability. This is in keeping with the mechanism of HIV associated injury which results in increased glial activation usually without significant neuronal abnormality until the disease is more advanced.

### Biochemical measures and MRS

Chemokines are multifunctional, immunomodulatory proteins that influence HIV neuropathogenesis by multiple mechanisms. In raised concentrations they are considered to be potentially neurotoxic. Several studies have shown the correlation between neurometabolites and chemokines (CXC chaemokine IP-10 and CC chaemokine MCP-1 (monocyte chemoattractant protein).

Metabolite improvement has been correlated with chemokines especially CSF MCP—monocyte chemoattractant protein (MCP-1) and IP-10. Higher MCP-1 levels are inversely associated with neuronal dysfunction (NAA and Glutamates) in untreated patients. After cART, MCP-1 is correlated with high glial response (increased Cho and Mi) rather than NAA. After 3 months of cART, the decreased systemic factors (viral burden, systemically derived MCP-1) were no longer associated with neuronal dysfunction, but subjects with the strongest glial response in the brain continue to produce the highest levels of MCP-1 [46]. Letendre et al. also identified higher levels of IP 10 correlating with reduced neuronal scores and higher basal ganglionic and inflammatory pattern scores by using advanced statistical paradigms [54].

*“These studies showed that the metabolite factor analysis method has a direct correlation with neuroinflammation and neuronal dysfunction as measured by chemokine pathway of IP 10 and MCP”*

### Excitotoxic injury and glutamatergic pathway

HAND is likely underpinned at least in part by neurotoxicity due to glial activation. Several recent studies have elaborated on this by showing the importance of Glu mediated excitotoxicity in the extracellular compartment. Glu is an amino acid which performs an important role in neurotransmission though when concentrations are high excitotoxic brain injury may occur. The possible mechanisms for increased extracellular glutamates include attenuation of astrocytic reuptake due to HIV infection and excessive production of Glu by HIV infected macrophages eventually leading to loss of the glutamates in the intracellular space [55, 56].

Several authors have demonstrated reduced intracellular Glx in the frontal white matter. Frontal grey matter and parietal grey matter and basal ganglia also showed abnormal Glx levels***.**** These studies showed a gradient in the reduction in Glx in NCS from MND to Dementia with increasing levels of reduced Glx with increasing neurocognitive impairment* [56, 57].

As stated earlier, study by Lentz et al, reduced Glu in the frontal cortical gray matter is a sensitive marker for identification of the CNS involvement by HIV virus [48].

Glx also appears to be sensitive to the effects of nucleoside reverse transcriptase inhibitors on the CNS. Reduced parietal grey matter Glx appeared to correlate with toxicity associated with nucleoside reverse transcriptase inhibitors [reduced astrocytic reuptake of Glu, secondary excitotoxicity, and mitochondrial toxicity from antiretroviral treatments [58].

*“The current evidence points to Glu may be not only useful as sensitive marker for very early neuronal injury, but also a good surrogate marker for disease severity and treatment effects”.*

### Patterns of pathological substrate: correlation between metabolites and HAND staging

The consequences of chronic HIV are multidimensional and the clinicopathological spectrum appears to be influenced not only by inflammation and neuronal loss but also by area of involvement. Mohammed et al. used these three patterns to prognosticate HAND staging with a specific pattern using advanced statistical algorithms [59].

Mohamed et al. employing factor analysis has identified three metabolic patterns: 1: the inflammatory factor.

(which was associated with mainly MI/Cr elevations in all three regions (BG, FWM and Parietal cortex) plus Ch/Cr increases in the centrum semiovale and parietal cortex), 2: the BG factor (associated with mostly NAA/Cr and Ch/Cr elevations in the BG), and 3: the neuronal factor (associated primarily with NAA/Cr reductions in the centrum semiovale and the parietal cortex). These factors were found to be useful in discriminating between the groups of cognitively impaired and unimpaired participants, with the neuronal pattern being strongly associated with HAND, that is ADC staging as it was known then [48, 60].

### MRS and comorbidities

Patients living with HIV have several comorbidities and characterisation of their contribution to NCI has been a challenge. Some of these comorbidities include aging, alcohol abuse, drug abuse and cART neurotoxicity.

### Aging

The increased life expectancy of the HIV positive individuals on cART has given rise to new challenges in characterising the cognitive impairment in this subgroup. Premature brain aging can be defined as greater than normal age-related deficits, but with similar or no greater rate of decline across the age spectrum, while accelerated brain aging can be demonstrated by a steeper rate of decline in brain measurements with age.

MRS studies performed during the pre-cART era or on antiretroviral-naive subjects suggest an accelerated aging process, while those on cART subjects suggest premature brain atrophy [61]. In normal aging there are changes in the metabolite concentrations in various regions of the brain with a slow steady increase in Mi and possibly some increase in the Ch and Cr [7] similar to HIV associated neural injury. However, HIV subjects who were naïve to cART showed greater than age-related decline in NAA and tCr in the BG and showed greater than age-related increases in Mi (+ 12% instead of 3%/decade) and Cho (+ 10% instead of 2%/decade) in the FWM, indicating that there is accelerated ageing in HIV disease [62]. Those who were on cART, showed premature ageing, with higher than normal Mi in the FWM across the age span and lower than normal tNAA/tCr in the FGM n the medial frontal cortex [59]. A multicentre study by Harezlak et al. [62] also reflected similar observations indicating that there is premature ageing. However, study by Cysique et al. have shown only a synergistic effect between the age and serostatus. Decreased NAA/unsuppressed water signal in frontal WM was associated with older age, particularly in the HIV + group. Ernst et al. observed decline in glutamate concentration with aging in parietal gray matter. In addition, this study also observed a trend for lower brain glutamate levels in the parietal and frontal cortex in HIV + individuals, more pronounced in individuals with cognitive impairment.

In conclusion, HIV related MRS abnormalities are variably worse with ageing. The MRS ageing changes in the cART era are more prominent with age, suggesting premature ageing. However, all these studies are cross sectional and may suffer from bias from intersubject variability. Longitudinal studies are needed.

### Alcoholism and HIV

“*Alcohol together with HIV appears to heighten the risk of neuronal loss in the brain*”.

Growing numbers of HIV patients abuse alcohol which is well known to be associated with accelerated brain aging [63]. One study comparing HIV + Alcohol and HIV -Alcohol groups using long TE MRSI demonstrated almost a full standard deviation reduction in NAA and Cr independent of cART status in the parietal–occipital region for the HIV + alcohol group [63]. On proton MRS, the Cho and Mi in the striatum were higher both in alcoholics with HIV and those who were not alcoholics and did not have any statistically significant change [64]. Although neither HIV infection nor alcoholism alone resulted in such a deficit, each disease carried a liability that put dually affected individuals at a heightened risk of neuronal compromise. These findings support the hypothesis that the alcohol has cumulative effect. Meyerhoff in pre cART era using 31P MRS observed that individuals with both conditions had augmented metabolite deficits but not interactive or synergistic.

### Methamphetamine effects of HIV (Meth)

“*Limited studies show that there is likely an additive effect on metabolites in patients with meth-abuse*.”

The effects of Meth on the brain are similar to HIV and the metabolic abnormalities are mainly localised to the frontal cortex, FWM and BG areas which are also typically involved in HIV. There are only a few studies available in patients with HIV with meth abuse, and these largely showed concordant results. The characteristic observations on MRS include markedly reduced NAA in both the BG and FWM and reduced Cr elevated Cho in the basal ganglia [64–66].

Metabolite changes in chronic meth users with HIV appears to be additive. HIV-negative subjects with a history of chronic meth use showed lower concentrations of the NAA in the FWM BG and higher concentrations of Cho compounds and Mi in the frontal cortex, relative to subjects with no history of drug abuse [66, 67].

However, Taylor et al. study concluded that there is likely no effect [68]. Although Taylor et al. argues that the increase in the volumes of the BG could have influenced the levels of the metabolites, it is difficult to explain how that may be so, given that the meth only group consistently demonstrated increased Cho in FWM and reduced NAA in the BG which are also observed in HIV [68].

### Antiretrovirals and NCI

Whether antiretrovirals can cause brain neurotoxicity is still in debate. Reduction in NAA secondary to mitochondrial injury may occur with Zidovudine (AZT) and other nucleoside reverse transcriptase inhibitors [69, 70]. Didanosine and Stavudine use has been associated with lower NAA when compared to the sero negative controls in the FWM [70]. However, a study using Glx as a marker did not show any differences in Glx between naïve and cART treated patients and concluded that the toxic effects of cART are therefore unlikely [71]. Nonetheless Ernst et al. identified reduced parietal gray matter Glu in HAND patients while normal in seronegative controls and opined that the resultant excitotoxic injury is secondary to NRTIs [58]. *“The likely cause for these differing observations may be secondary to differing cART regimens as well as individual host susceptibility. The reduction in the Glu could be secondary to reduced astrocyte reuptake of Glu, secondary excitotoxicity, and mitochondrial toxicity from antiretroviral treatments”.*

## Conclusion

MRS can be a useful biomarker to measure brain injury in HIV and is likely to guide understanding of neurocognitive impairment at the cellular level. In the setting of cognitive decline, the MRS findings are increased Ch and Mi both in the FWM and BG early on and in the later stages there is compromise of the neuronal integrity signalled by reduced NAA. Creatine concentrations can change depending on the severity of dementia and therefore concentrations either absolute or relative to internal water are more sensitive. Although well established as early as in 2002 by Chang et al., multiple studies thereafter have noted the metabolite changes based on creatine; this is perhaps an issue that should be incorporated into guidelines. MRS changes correlating with cART improvement can take as long as 6–12 months. However, recent evidence with measurement of the excitotoxic pathway using as Glx and neuroinhibitory pathway using GABA have shown that they are the earliest to be identified in MRS [42].

A recent metaanlysis by Chelala et al. on a pooled study of 61 spectroscopic studies concluded that there is consistently lower tNAA/tCr, higher tCh/tCr and higher mI/tCr ratios associated with chronic HIV infection. However, this study does acknowledge that the Creatine contribution needs to be assessed independently and interpretation is difficult because of varying treatments, duration of infection, and comorbidities [72].

In summary then MRS is a useful tool in HIV infection to assist in HAND identification, response to cART, and insight into neuropathogenesis.

## Data Availability

Not applicable.

## Appendix: MRS studies in HAND

**Table:**

Study	Journal	Model	MRS-tech and area of exploration	Conclusions
Chang L, Ernst T et al. Relationships among brain metabolites, cognitive function, and viral loads in antiretroviral-naıve HIV patients	Neuroimage. 2002;17(3):1638–48	Relationship of metabolites and cognitive function and clinical variables	1.5 T-PRESS (30 TE): FWM,BG, FGM Voxel: NA	Frontal lobe [MI], [CHO], and total creatine [CR] were elevated, while basal ganglia [CR] were decreased with increasing dementia severity in turn suggesting that the concentrations but not the ratios should be measured
Paley M, et al. A multicenter proton magnetic resonance spectroscopy study of neurological complications of AIDS	AIDS research and human retroviruses. 1996;12(3):213–22	Correlation between the metabolites and clinical variables	1.5 T Long TE MRS- PO WM Single Voxel Voxel: 8^3^ Cm	In HIV patients, there was no significant correlation between metabolite ratios of brain detected by MRS and CDC grouping of patients or CD4 count. In contrast, the variations reduced NA/Cr, NA/Cho, and increased Cho/Cr) were related to the occurrence of encephalopathy, brain atrophy, or diffuse white matter lesions
Chang L, Ernst T, et al. Cerebral metabolite abnormalities correlate with clinical severity of HIV-1 cognitive motor complex	Neurology. 1999;52(1):100–	biochemical alterations and disease severity in HIV cognitive motor complex (HIV-CMC)	1.5 T FGM, FWM, BG Voxel: 3–5^3^ Cm	HIV-CMC patients had elevated MI and CHO levels with increasing AIDS dementia complex stage, and *N*-acetyl compounds (NA) were decreased only in moderate to severe stages of dementia
Salvan AM, et al. Brain proton magnetic resonance spectroscopy in HIV-related encephalopathy	AIDS research and human retroviruses. 1997;13(12):1055–66	Metabolites and NAS seropositive	1.5 T: LONG TE MRS-SV PW WM Voxel: 2^3^ Cm	NAS and ADC increased choline and decreased NAA, with NAA Decrease more conspicuous in ADC. Improvement of the metabolites with ART
Jarvik JG, Lenkinski RE, et al. Proton MR spectroscopy of HIV-infected patients: characterization of abnormalities with imaging and clinical correlation	Radiology. 1993;186(3):739–44	Metabolites and ADC Stages comparison	1.5 T: short TE, abnormal WM Voxel: 2 cm^3^	Increased choline/creatine ratio and increased Marker peak (Glx)
Tracey ID, et al. Brain choline-containing compounds are elevated in HIV-positive patients before the onset of AIDS dementia complex: a proton magnetic resonance spectroscopic study	Neurology. 1996;46(3):783–8	Metabolites and staging of ADC,	Long TE -Parieto occipital Gray 2 cm^3^	Increased choline/creatine ratio and reduced NAA/Cr and with increasing severity of cognitive decline increase in the Choline and decrease in NAA
Barker PB, Lee RR, McArthur JC. AIDS dementia complex: evaluation with proton MR spectroscopic imaging	Radiology. 1995;195(1):58–64	Metabolites and ADC Staging	Long TE (270)-multivoxel multiple slices-NWM 32/32 Voxels/s	Increased choline/creatine ratio and reduced NAA/Cr and with increasing severity of cognitive decline increase in the Choline and decrease in NAA
Chang L, Ernst T, Leonido–Yee M, Witt M, Speck O, Walot I, et al. Highly active antiretroviral therapy reverses brain metabolite abnormalities in mild HIV dementia	Neurology. 1999;53(4):782	To determine cerebral metabolite abnormalities, normalize with HAART	1.5 T.FWM and BG Short TE Voxel: N/A	HAART improves HIV-CMC in addition to systemic measures of HIV infection. Myoinositol Improvement corresponds with clinical recovery
Meyerhoff D, Bloomer C, Cardenas V, Norman D, Weiner M, Fein G. Elevated subcortical choline metabolites in cognitively and clinically asymptomatic HIV patients	Neurology. 1999;52(5):995–	To determine the relationship of NAS and metabolites NAA and Choline in Subcortical brain	1.5 T: Long TE (135) FWM and BG Voxel: 15 mm^3^	^1^H MRS imaging detects higher Cho in subcortical brain early in HIV disease, when individuals are clinically and neuropsychologically asymptomatic, whereas lower NAA is only found in subcortical brain in individuals with severe neuropsychological impairment
Laubenberger J, HIV-related metabolic abnormalities in the brain: depiction with proton MR spectroscopy with short echo times	Radiology. 1996;199(3):805–10	To measure the changes in metabolites in NAS vs HIV ADC	2 T: Short TE-Parieto occipital WM Voxel: 2.5 cm^3^	MRS abnormalities are seen in both the groups but more significant in the ADC. Reduced NAA/Cr, increased Mi/Cr and increased Cho/Cr
Menon DK, Proton MR spectroscopy and imaging of the brain in AIDS: evidence of neuronal loss in regions that appear normal with imaging. tomography	JCAT. 1990;14(6):882–5.1990;14(6):882–5	Metabolites in AIDS Dementia complex-first report	0.15 T-Long TE Parietal cortex and white matter Voxel: 4 cm^3^	Low NAA/Cr and High Choline/Cr ratio
Chong W, et al. Proton spectroscopy of the brain in HIV infection: correlation with clinical, immunologic, and MR imaging findings	Radiology. 1993;188(1):119–24	Metabolites with early and late stages of human immunodeficiency virus (HIV) infection	1.5 T, Long TE OCCIPITAL WM Voxel 2 cm3	NAA as putative neuronal marker. Low in AIDS seropositive vs Normal controls as well as in CMC > normal controls
Meyerhoff DJ, et al. Reduced brain N‐acetylaspartate suggests neuronal loss in cognitively impaired human immunodeficiency virus‐seropositive individuals: spectroscopic imaging	Neurology. 1993;43(3 Part 1):509	Early neurocognitive impairment (Mild) and MR metabolites sensitivity	1.5 Short TE supra ventricular WM CSI-7 voxels	NAA as putative neuronal marker. Low in Early CI as compared to the normals
Chang L, Ernst T, Witt MD, Ames N, Walot I, Jovicich J, et al. Persistent brain abnormalities in antiretroviral-naive HIV patients 3 months after HAART	Antiviral therapy. 2003;8(1):17–26	Longitudinal: Metabolites and cognitive function before and after three months of cART	1.5tShort TE FC, FWM, BG Voxel: 2 cm^3^	Persistent metabolite abnormalities after three months but returned back to normal after 9 months
Tarasow E, et al. Cerebral MR spectroscopy in neurologically asymptomatic HIV-infected patients	Acta Radiologica. 2003;44(2):206–12	Metabolites and NAS	1.5 T Short TE FWM Voxel: 2 cm^3^	**Reduced CR/H2O** and reduced NAA/H2O and reduced NAA/Cho as well as elevated Mi and Cho to water. Reduced Energy metabolism in HIV-Asymptomatic
Yiannoutsos CT, Nakas CT, Navia BA, Consortium PM. Multidimensional ROC and: application to proton MR Spectroscopy (MRS) in HIV-related neurological injury	Neuroimage. 2008;40(1):248–55	Multi-dimensional receiver operating characteristic statistical algorithm	1.5 T Short TE-PC, FWM and BG Voxel 6 Cm^3^	Highest sensitivity in asymptomatic individuals for MI/Cr and Cho/Cr in the BG and NAA/Cr in the FWM as compared with the controls: Continual of CNS injury with HIV infection.: NAA/Cr decreases in ADC group, raising issues of synergism between HIV infection and age and possible acceleration of neurological deterioration in an aging HIV-positive population
Tarasów et al.: Antiretroviral therapy and its influence on the stage of brain damage in patients with HIV – 1H MRS evaluation	Med Sci Monit, 2004; 10(Suppl 3): 101–106	Long: Influence on the Metabolites in clinically asymptom HIV-infected	1.5 T. Long Study: on average after 6 months. FWM Voxel: 2 cm^3^	HAART affects clinically asymptomatic HIV + patients and diminishes the risk of ADC occurrence Myoinositol and choline levels indices for antiretroviral treatment efficacy
Chang L, Speck O, Miller EN, Braun J, Jovicich J, Koch C, et al. Neural correlates of attention and working memory deficits in HIV patients	Neurology. 2001;57(6):1001–7	Functional MRI only	N/A	N/A
Stankoff B, Tourbah A, Suarez S, Turell E, Stievenart J, Payan C, et al. Clinical and spectroscopic improvement in HIV-associated cognitive impairment	Neurology. 2001;56(1):112–5	Longitudinal study: 9 months HAART and response Metabolites	1.5 T: Short TE -Centrum semiovale WM and Occipital GM Voxel: 2 cm^3^	Neurologically intact no changes. And In impaired partial response in NAA/Cr and Cho/Cr
Wilkinson ID, et al. Proton MRS and quantitative MRI assessment of the short term neurological response to antiretroviral therapy in AIDS	JNNP. 1997;63(4):477–82	Longitudinal study: 4 weeks HAART	1.5 T. Long TE Parieto occipital white matter Multiple voxels	increases in *N*-acetyl/(*N*-acetyl + choline + creatine) ratio and neurological status deteriorated the NA/(NA + Cho + Cr)
Chang L, Jiang C, Cunningham E, Buchthal S, Douet V, Andres M, et al. Effects of APOE ε4, age, and HIV on glial metabolites and cognitive deficits	Neurology. 2014;82(24):2213–22	Cross sectional-Impact of APOE e4 on glial metabolites	1.5 T, BG, parietal GM, FWM and anterior cingulate GM Voxel: 2 cm^3^	The combined effects of HIV infection and APOE e4 may lead to greater cognitive deficits, -Synergistic
Young AC, Yiannoutsos CT, Hegde M, Lee E, Peterson J, Walter R, et al. Cerebral metabolite changes prior to and after antiretroviral therapy in primary HIV infection	Neurology. 2014;83(18):1592–600	Long study: 6 months effect of cART	1.5 T. BG, parietal GM, FWM and anterior cingulate GM Voxel: 2 cm^3^	Increased choline/creatine and increased Mi/Cr and Increased Glu/Cr in the treatment Naïve and post treatment Glu/Cr ratio reduction
Sailasuta N, et al. Neuronal-glia markers by magnetic resonance spectroscopy in HIV before and after combination antiretroviral therapy	Journal of acquired immune deficiency syndromes 2016;71(1):24	Long study: 12 M, Chronic HIV and effect of cART	BG, parietal GM, FWM and Posterior cingulate Voxel: 2 cm3	cART is associated with reduced neuronal-glia and inflammatory markers. Alterations in CHO are noted among individuals who remain impaired after 12 months of cART
Cysique LA, Jugé L, Gates T, Tobia M, Moffat K, Brew BJ, et al. Covertly active and progressing neurochemical abnormalities in suppressed HIV infection	Neurology-Neuroimmunology Neuroinflammation. 2018;5(1)	Long study: 12 M, Chronic HIV and effect of cART	3 T. BG, FWM and Posterior cingulate Voxel: 2 cm3	covertly active or progressing HIV-related brain injury in the majority of this virally suppressed cohort Creatine and NAA showed decrease in the progressive HAND in the PCC
Boban J, et al. neurometabolic Remodelling in chronic HIV infection: a five-Year follow-up Multi-Voxel Mrs Study	Scientific Reports. 2019;9(1):1–11	Long study: 5 years follow up Aviremic HIV on cART	FC, PC intraparietal sulci, and FWM Multiple Voxels	Increase in NAA/Cr ratio with progressive improvement
Gongvatana A, Harezlak J, Buchthal S, Daar E, Schifitto G, Campbell T, et al. Progressive cerebral injury in the setting of chronic HIV infection and antiretroviral therapy	Journal of neurovirology. 2013;19(3):209–18	Long study: 2 years Neurocognitive normal and aviremic	1.5 T. MFC, FWM, BG NAA, Cr, Cho, Mi and Glx. (multiple sites) Voxel: 2 cm^3^	Neuronal injury, occur in chronically HIV-infected persons despite stable antiretroviral treatment and virologic suppression and can lead to neurocognitive declines. Neurocognitive decline was associated with longitudinal decreases in Glx in the FWM and the BG, and in NAA in the BG
Robertson K, Su Z, Margolis D, Krambrink A, Havlir D, Evans S, et al. Neurocognitive effects of treatment interruption in stable HIV-positive patients in an observational cohort	Neurology. 2010;74(16):1260–6	No MRS		
Chang L, Ernst T, St Hillarie C, Conant K. Antiretroviral treatment alters relationship between MCP-1 and neurometabolites in HIV patients	Antiviral therapy. 2004;9(3):431–40	Long study: The relationships between neurometabolites and MCP-1 in serum and CSF	1.5 T. 3 months follow up voxels, 3–5 ml^3^	Higher CSF MCP-1 levels are associated with neuronal dysfunction in untreated patients. After 3 months of HAART no longer associate with neuronal dysfunction
Suwanwelaa N, et al. Magnetic resonance spectroscopy of the brain in neurologically asymptomatic HIV-infected patients	Magnetic resonance imaging. 2000;18(7):859–65	MRS early brain involvement in neurologically asymptomatic HIV-infected patients	1.5 T. centrum semiovale and Thalamus Voxel: 2.2 cm^3^	Significant reduction in NAA/Cr and NAA/Cho in both centrum semiovale and thalamic areas. MRS is more sensitive than conventional MR imaging. No statistically significant difference as to Cho)/Cr and mI/Cr ratios in both regions
Paul R, Cohen R, Navia B, Tashima K. Relationships between cognition and structural neuroimaging findings in adults with human immunodeficiency virus type-1	Neuroscience and Biobehavioral Reviews. 2002;26(3):353–9	Review		
Avison MJ, Nath A, Berger JR. Understanding pathogenesis and treatment of HIV dementia: a role for magnetic resonance?	Trends in neurosciences. 2002;25(9):468–73	Review		
Mohamed MA, et al. Brain metabolism and cognitive impairment in HIV infection: a 3-T magnetic resonance spectroscopy study	Magnetic resonance imaging. 2010;28(9):1251–7	GLx assessment in three groups (Cognition: Normal-MCI and HAD	3 T. FWM Voxel: 2.2 cm^3^	Glx/Cr is reduced with increasing CI. Associated with impaired performance in specific cognitive domains. Glx may be a useful indicator of neuronal loss/dysfunction in patients with HIV infection
Lentz M, Kim W, Lee V, Bazner S, Halpern E, Venna N, et al. Changes in MRS neuronal markers and T cell phenotypes observed during early HIV infection	Neurology. 2009;72(17):1465–72	Assessment of metabolites in early HIV infection (60 days of seroconversion)	1.5 T FC and Centrum Semiovale	Lower NAA and Glx levels in the cortical gray matter suggests that HIV causes neuronal dysfunction soon after infection- correlates to the expansion of CD8 T cells. Tracking NAA levels may provide understanding of the virus–host interactions involved in CNS functional deficits
English CD, et al. Elevated frontal lobe cytosolic choline levels in minimal or mild AIDS dementia complex patients: a proton magnetic resonance spectroscopy study	Biological psychiatry. 1997;41(4):500–2	characterization of FC changes throughout the disease process for evaluating both disease progression and treatment	1.5 T Frontal cortex 6 cm^3^	ADC-Increased Co/Cr ratio is the single most specific observation
von Giesen H-J, Basal ganglia metabolite abnormalities in minor motor disorders associated with human immunodeficiency virus type 1	Archives of neurology. 2001;58(8):1281–6	psychomotor speed, and metabolic alterations in the BG in MMD	1.5 T BG- Short TE Voxel: 3 cm^3^	No change in patients with incipient HIV-1 MMD, -sustained HIV-1 MMD showed elevated Mi/CR incipient HIV-1 MMD -no metabolite changes in the BG, patients with sustained HIV-1 MMD did have significantly altered metabolic spectra indicative of glial proliferation
López-Villegas D, Lenkinski RE, Frank I. Biochemical changes in the frontal lobe of HIV-infected individuals detected by magnetic resonance spectroscopy	Proceedings of the National Academy of Sciences. 1997;94(18):9854–9	Metabolites and ADC stages comparison	1.5 T Frontal cortex	ADC-Normal Mi/Cr and reduced NAA/Cr whilst those without ADC have increased Mi/Cr
Sacktor N, et al. Combination antiretroviral therapy improves psychomotor speed performance in HIV-seropositive homosexual men	Neurology. 1999;52(8):1640-	Only clinical measures and No MRS		
Paul RH, et al. Proton MRS and neuropsychological correlates in AIDS dementia complex: evidence of subcortical specificity	The Journal of neuropsychiatry and clinical neurosciences. 2007;19(3):283–92	MRS and NPS with AIDS dementia complex (stages 1–3) correlation	1.5 T.BG, FWM, and parietal cortex Multisite study (Voxel N/A)	Neuropsychological impairment is associated with reduced markers of mature neurons and increased markers of gliosis in the basal ganglia and frontal white matter. (Higher NAA with higher NPZ in the FWM and reduced NPZ with increased Mi in the BG
Schifitto G, et al. Memantine and HIV-associated cognitive impairment: a neuropsychological and proton magnetic resonance spectroscopy study	Aids. 2007;21(14):1877–86	Longitudinal study: safety and efficacy of memantine	F1.5 T. WM and Parietal cortex Voxel: 6 Cu ml	No improvement in the NPZ -8 but increased NAA/Cr ratio in both the FWM and Parietal cortex
Paul RH, et al. Relative sensitivity of magnetic resonance spectroscopy to cognitive function among nondemented individuals infected with HIV	Journal of the International Neuropsychological Society. 2008;14(5):725–33	Relationships among NCF-Metabolites-in BG and Volumetry of the CN and Putamen in the earliest stages of HAND	1.5 T. NPZ-VOLUMETRY OF CN and LN and MRS of Caudate nucleus 6 Cu ml	MRS differences are more significant that the volumetry in mild stages of HIV-HAND
Cohen RA, et al. Cerebral metabolite abnormalities in human immunodeficiency virus are associated with cortical and subcortical volumes	Journal of neurovirology. 2010;16(6):435–44	Relationship between the metabolite disturbances would be associated with reduced cortical and subcortical volumes	1.5 T. FGM, FWM and BG-MRS AND Volumetry 6 Cu ml	Reduced cortical and subcortical volumes, are also strongly associated with the degree of cerebral metabolite disturbance (↓ NAA- Mid frontal cortex,↓ Glx-Temporal Lobe
Ernst T, Chang L, Leonido–Yee M, Speck O. Evidence for long-term neurotoxicity associated with methamphetamine abuse: a 1H MRS study	Neurology. 2000;54(6):1344–9	proton MRS and metabolite abnormalities in abstinent methamphetamine users	1.5 T. FC, FWM, BG 2 Cm^3^	Reduced NAA, Increased Mi and Choline as well as reduced Creatine
Cloak C, Increased frontal white matter diffusion is associated with glial metabolites and psychomotor slowing in HIV	Journal of neuroimmunology. 2004;157(1–2):147–52	Diffusion imaging and MRS metabolites correlation	1.5 T FWM and BG Voxel: N/A	Diffusivity correlated positively with MI and negatively with cognitive performance
Schifitto G, Zhong J, Gill D, Peterson DR, Gaugh MD, Zhu T, et al. Lithium therapy for human immunodeficiency virus type 1–associated Ernst T, neurocognitive impairment	Journal of neurovirology. 2009;15(2):176–86	Longitudinal (10 weeks)lithium safety and tolerability and its impact on cognition, function and in HIV -MRS,rs-fMRI, DTI	1.5 T FGM and FWM and BG Voxel: 2 Cm^3^	Decreased GLX and Increased FA and Increased connectivity. Suggests lithium may improve HIV-associated CNS injury
Chang L, Arnold S. Increased glial metabolites predict increased working memory network activation in HIV brain injury	Neuroimage. 2003;19(4):1686–93	Attentional deficits in early HIVAIDS Dementia complex stage 1 and cause as brain inflammation MRS and fMRI	1.5 T the frontal gray and white matter, and basal ganglia. And Functional MRI Voxel: 2 Cm^3^	Mild HIV brain injury is associated with increased glial activation without major involvement of neuronal abnormalities. ( increased Choline, Creatine and Mi) reduced activation in the visual cortex
Letendre SL, Zheng JC, Kaul M, Yiannoutsos CT, Ellis RJ, Taylor MJ, et al. Chemokines in cerebrospinal fluid correlate with cerebral metabolite patterns in HIV-infected individuals	Journal of neurovirology. 2011;17(1):63–9	Correlation of Chaemokines and Cerebral metabolites in HIV-HAND	1.5 T. BG, parietal GM, FWM AND FGM Voxel: 2 Cm^3^	Higher IP-10 levels correlated with lower NAA)/creatine (Cr) ratios in FWM and higher MI/Cr ratios in all three brain regions. Higher MCP-1 = Lower NAA/Cr. antiretroviral therapy and memantine modify the impact of the immune response on neurons
Sailasuta N, Ernst T, Chang L. Regional variations and the effects of age and gender on glutamate concentrations in the human brain	Magnetic resonance imaging. 2008;26(5):667–75	Effect of age on Glx	3 T. BG, FGM, PGM Voxel sizes: 8 cc: the FWM, FGM and PGM and 12 cc for the BG	Glu concentration in the GM was approximately 25% higher than that in the WM Glu concentration in the GM was approximately 25% higher than that in the WM
Chang L, Cloak C, Yakupov R, Ernst T. Combined and independent effects of chronic marijuana use and HIV on brain metabolites	Journal of Neuroimmune Pharmacology. 2006;1(1):65–76	Chronic MJ use and HIV infection are associated with interactive or additive effects on brain chemistry and cognitive function	4 T. thalamus and the BG (all 96) subjects; in addition, FWM (n = 56), cerebellar vermis (n = 47), right parietal white matter (n = 49), and occipital gray matter (n = 45) Voxel: 3–5 ml	Chronic MJ use may lead to decreased neuronal and glial metabolites, but may normalize the decreased glutamate in HIV patients
Ernst T, Lower brain glutamate is associated with cognitive deficits in HIV patients: A new mechanism for HIV‐associated neurocognitive disorder	Journal of Magnetic Resonance Imaging. 2010;32(5):1045–53	Reduced conc. of GLU, and whether lower GLU levels correlate with cognitive deficits	3 T. BG, parietal GM, FWM, and Anterior Cingulate GM Voxel: 8 cc	Parietal gray matter GLU is lower in HIV subjects with cognitive deficits. The glutamatergic system may play an important role in the pathophysiology of HAND, and brain GLU on 1H MRS may provide an early surrogate marker for monitoring disease severity and treatment effects
Holt JL, Kraft-Terry SD, Chang L. Neuroimaging studies of the aging HIV-1-infected brain	Journal of neurovirology. 2012;18(4):291–302	Review	N/A	N/A
Ernst T, Chang L. Effect of aging on brain metabolism in antiretroviral-naive HIV patients	Aids. 2004;18:61–7	MRS consortium: to evaluate the neurometabolites in HIV patients with or without cognitive impairment	Multi-centre trial BG, FWM, PC Voxel: 2 cm^3^	Inflammatory activity in the BG and neuronal injury in the white matter is associated with the development of cognitive impairment Aging may further exacerbate brain metabolites associated with inflammation in HIV patient and thereby increase the risk for cognitive impairment
Harezlak J, et al. Persistence of HIV − associated cognitive impairment, inflammation and neuronal injury in era of highly active antiretroviral treatment	AIDS (London, England). 2011;25(5):625	1.5 T whether cognitive impairment and brain injury as measured by MRS persist in the setting of HAART	1.5 T FWM, BG, PC Voxel: 2 cm3	Brain inflammatory changes remain ubiquitous among HIV-infected individuals, whereas neuronal injury occurs predominantly in those with cognitive impairment Together these findings indicate that despite the widespread use of HAART, HIV-associated cognitive impairment and brain injury persist in the setting of chronic and stable disease
Pfefferbaum A, Adalsteinsson E, Sullivan EV. Cortical NAA deficits in HIV infection without dementia: influence of alcoholism comorbidity	Neuropsychopharmacology. 2005;30(7):1392–9	Assess the combined effects of Alcoholism and HIV on the brain function	1.5 T MRSI Long TE Multiple voxels	Additive effect
Zahr NM, Mayer D, Rohlfing T, Sullivan EV, Pfefferbaum A. Imaging neuroinflammation? A perspective from MR spectroscopy	Brain pathology. 2014;24(6):654–64	Review		
Nordahl TE, et al. Low N-acetyl-aspartate and high choline in the anterior cingulum of recently abstinent methamphetamine-dependent subjects: a preliminary proton MRS study	Psychiatry Research: Neuroimaging. 2002;116(1–2):43–52	N/A		
Nordahl TE, Salo R, Natsuaki Y, Galloway GP, Waters C, Moore CD, et al. Methamphetamine users in sustained abstinence: a proton magnetic resonance spectroscopy study	Archives of general psychiatry. 2005;62(4):444–52	N/A		
Sekine Y, et al. Metabolite alterations in basal ganglia associated with methamphetamine-related psychiatric symptoms: a proton MRS study	Neuropsychopharmacology. 2002;27(3):453–61	N/A		
Taylor MJ, GROUP H. MR spectroscopy in HIV and stimulant dependence	Journal of the International Neuropsychological Society. 2000;6(1):83–5	MRS: to examine the additive effects of HIV infection and stimulant dependence on frontostriatal circuitry	1.5 T FWM, CSO, CN 2.0 CM^3^	Raise the possibility that stimulant dependence may potentiate HIV related neuronal injury,
Taylor M et al.: Effects of human immunodeficiency virus and methamphetamine on cerebral metabolites measured with magnetic resonance spectroscopy	J Neurovirol. 2007 Apr;13(2):150–9	HIV + methamphetamine effects on virus	1.5 T FWM, FGM, BG	Significant disruption of neuronal integrity in the frontal lobes of HIV-infected individuals. Although METH was not associated with cerebral metabolite levels, other findings suggested that METH use did affect the brain
Winston A, et.al Does choice of cART alter changes in cerebral function testing after 48 weeks in treatment-naïve, …. HIV-1 cART? A randomized, controlled study	Clinical infectious diseases. 2010;50(6):920–9	Differences between changes in cerebral function and alternative cARTs have not been prospectively assessed	3.0 T and 1.5 T Cn, FWM, FGM 2 Cm^3^	Differential improvement with different drug regimens in NAA/Cr ratios
Schweinsburg BC, et al. Brain mitochondrial injury in human immunodeficiency virus-seropositive (HIV +) individuals taking nucleoside reverse transcriptase inhibitors	Journal of neurovirology. 2005;11(4):356–64	NRTIs suppress HIV replication, but with mitochondrial toxicity	1.5 T FWM and gray mater	Reductions in NAA in individuals taking didanosine and/or stavudine may be the result of depleted brain mitochondria and/or alterations in cellular respiration. Measurement of brain metabolites sensitive to impairments in energy metabolism, including NAA, may aid in early detection of subclinical NRTI-mediated mitochondrial toxicity
Bladowska et al.: Evaluation of metabolic changes within the normal appearing gray and white matters in neurologically asymptomatic HIV-1-positive and HCV-positive patients: Magnetic resonance spectroscopy and immunologic correlation	European Journal of Radiology 82 (2013) 686–692	To evaluate early metabolic changes using proton MR spectroscopy (MRS) in asymptomatic HIV-1-positive and HCV-positive patients without abnormalities in the structural MR examination	1.5 T PWM, FWM, BG, AC, PC	Significant decrease (p < 0.05) of the NAA/Cr ratios in PCG, ACG and PWM regions in HIV-1-positive cART treated patients compared to the normal subjects The metabolic changes—reduction of NAA/Cr ratios are most pronounced in HIV-1-positive patients using cART. The low CD4n T cell count is a risk factor for neurocognitive impairment in HIV-1-positive patients

## Abbreviations

MRS: MR spectroscopy, MR spectroscopic associated terminology
NAA: *N* Acetyl aspartate
Cho: Choline
Cr: Creatine
CrT: Total creatine
Mi: Myoinositol
GABA: Gamma amino butyric acid
Glx: Glutamine and glutamate
Glu: Glutamine
Glt: Glutamates
MRSI: MR spectroscopic imaging
PRESS: Point RESolved spectroscopy
STEAM: Stimulated echo acquisition mode
TR: Time to repeat
TE: Time to echo
